# Perinephric fat volume and its negative correlation with the eGFR according to a population health screening: a cross-sectional study

**DOI:** 10.1080/0886022X.2025.2516208

**Published:** 2025-06-12

**Authors:** Xiaohui Zhang, Yaqing Zhou, Dele Bo, Jia Liu, Xiaoran Cui, Zuoyang Zhang

**Affiliations:** The Second Hospital of Hebei Medical University, Shijiazhuang, Hebei, P. R. China

**Keywords:** Perinephric fat volume, estimated glomerular filtration rate, CKD, cross-sectional study

## Abstract

**Background:**

Chronic kidney disease (CKD) affects 10% of the global population. This study investigated the correlation between ultrasonographically assessed perinephric fat volume (PrFV) and the estimated glomerular filtration rate (eGFR) through a health screening of a Chinese population and explored the potential of the PrFV as a modifiable risk factor for CKD.

**Methods:**

This cross-sectional study included 213 adults aged 18 years or older from the Second Hospital of Hebei Medical University. Demographic data, medical history, the results of anthropometric assessments, and laboratory data were collected. The PrFV was measured using ultrasonography. Univariate and multivariate regression models were used to assess the association between the PrFV and the eGFR.

**Results:**

The median age of the participants was 45.8 years, with a median eGFR of 83.6 mL/min/1.73 m^2^. Compared with the lowest PrFV tertile group, the highest PrFV tertile demonstrated significantly lower eGFRs. Additionally, body weight and BMI were greater in the highest PrFV tertile group (*p* < 0.001). Multivariate linear regression revealed a significant negative association between the PrFV and the eGFR (β=-1.30, 95% CI −1.87 to −0.74, *p* < 0.001), indicating that each 10 cm³ increase in the PrFV corresponds to a 1.30 mL/min/1.73 m^2^ decrease in the eGFR, after adjusting for potential confounders.

**Conclusion:**

There was an independent negative correlation between the PrFV and the eGFR according to a population health screening. These findings suggest that an increase in the PrFV is associated with a decrease in the eGFR, warranting further investigation into the mechanistic role of perinephric fat in CKD.

## 1. Introduction

Chronic kidney disease (CKD) represents an increasing global health challenge, impacting an estimated 10% of the global population [1]. According to a joint statement from key nephrology organizations, as of 2021, kidney disease affects more than 850 million individuals, roughly twice the number of individuals affected by diabetes (422 million) and 20 times the number of people affected by cancer globally (42 million) and significantly exceeding the number of HIV/AIDS cases, which is estimated at 36.7 million [2]. Conditions such as obesity, hypertension, and diabetes significantly increase the risk of kidney disease, with diabetes and hypertension predominantly driving chronic kidney damage, accounting for more than 70% of cases of end-stage renal disease [3,4]. With societal advancements, the prevalence of obesity continues to increase annually, increasing concerns about obesity-related chronic kidney disease. Recent research indicates a ten- to twenty-fold increase in the incidence of obesity-related kidney damage compared with historical levels [5,6]. Body mass index (BMI), waist circumference (WC), and traditional obesity metrics are correlated with disease progression in all stages of CKD [7–9]. Numerous observational studies have revealed a correlation of a high BMI with impaired renal function [10–12]. The interplay between obesity and CKD is well established, with obesity influencing both the onset and progression of CKD, potentially through chronic inflammation, heightened oxidative stress, and hyperinsulinemia [13–15].

Previous studies have explored the relationships between traditional indicators of obesity, such as BMI, WC, and the waist-to-hip ratio (WHR), with CKD. However, these traditional indicators have limitations in differentiating fat from other tissue components and in specifying distinct fat distributions. Moreover, they are influenced by age, ethnicity, and physical activity levels. In a study involving 1,261 middle-aged Norwegians without diabetes, cardiovascular disease, or kidney disease, neither BMI nor the WHR was associated with an annual decline in the estimated glomerular filtration rate (eGFR) [16]. These findings suggest that traditional indicators of obesity, such as BMI, the WC, and the WHR, may have limitations in analyses of the relationship between obesity and CKD. With the advancement of medical imaging technologies, new methods for identifying adipose tissue have emerged in clinical practice and scientific research. Numerous studies have indicated that visceral fat is more closely related to CKD [17–19]. Among the types of visceral fat, perirenal fat is particularly involved in energy metabolism and the secretion of adipokines. Anatomical studies have shown that, unlike traditional connective tissue, perirenal fat has a complete blood supply and lymphatic drainage and is innervated [20]. Histologically, pararenal fat is primarily composed of white adipose tissue, whereas perirenal fat is a neutral fat consisting of white adipose tissue and islands of brown adipose tissue [21]. Compared with other types of visceral fat, perirenal fat is more strongly involved in energy metabolism and adipokine secretion [22]. Adipokines are cytokines that can regulate renal function through paracrine or endocrine pathways. Regardless of BMI, perirenal fat is associated with renal dysfunction in hypertensive patients [23]. Multiple studies have confirmed that, in populations with hypertension and diabetes, perirenal fat is a better predictor of creatinine clearance than traditional obesity indicators and other types of visceral fat [24,25].

In previous studies, the thickness of specific perinephric fat areas was measured using ultrasound, which is insufficient for quantifying the amount of perinephric fat. Given the heterogeneous distribution of perinephric fat, particularly its relatively high concentration in the lower regions, we applied longitudinal, transverse, and anteroposterior ultrasound of lower perinephric fat, thereby increasing the accuracy and reliability of perinephric fat volume (PrFV) quantification [26,27]. We subsequently conducted a cross-sectional study to explore the association between the PrFV and eGFR through a health screening of a Chinese population.

## 2. Methods

### 2.1. Study population

This study included 213 participants who underwent health checkups at the Second Hospital of Hebei Medical University from August 2023 to January 2024. All the participants were at least 18 years of age. Their demographic information and medical history were collected by physicians who had undergone specialized training.

The inclusion criteria were as follows: no history of diabetes, no history of hypertension, no cardiovascular disease (including heart failure), no prior acute kidney disease, no prior acute kidney injury, no history of chronic kidney disease (defined as an eGFR < 60 mL/min/1.73 m^2^), and no history of renal surgery.

The exclusion criteria were as follows: positive urine protein, elevated urine red blood cell count, abnormal renal morphology or number, use of medications to reduce creatinine levels or undergoing renal replacement therapy (transplantation or dialysis), living in areas with a high prevalence of apolipoprotein L1 genetic variants, structural urinary tract disease, recurrent kidney calculi, systemic lupus erythematosus, vasculitis, HIV, drug-induced nephrotoxicity and radiation nephritis, family history or known genetic variants associated with CKD, occupational exposures that promote CKD risk, cancer, and pregnancy.

The study protocol was approved by the Institutional Review Board of the Second Hospital of Hebei Medical University and was conducted in accordance with the principles of the Declaration of Helsinki. Written informed consent was obtained from each participant.

### 2.2. Anthropometric assessment

All the participants were dressed in light clothing without shoes, and their heights and weights were measured using a KF-1328LED body height and weight scale (Kaifeng Group Co., Ltd., Zhejiang, China). BMI was calculated in units of kilograms per square meter. WC was measured at the midpoint between the lower edge of the ribs and the highest point of the iliac crest.

### 2.3. Laboratory measurements

Systolic blood pressure (SBP) and diastolic blood pressure (DBP) were measured by experienced technicians using automatic blood pressure monitors. An SBP ≥ 140 mmHg or DBP ≥ 90 mmHg was defined as hypertension. Blood samples were collected after at least 8 h of fasting. The levels of total cholesterol (TC), high-density lipoprotein cholesterol (HDL-C), low-density lipoprotein cholesterol (LDL-C), triglycerides (TG), fasting plasma glucose (FPG), serum creatinine (SCr), and serum uric acid (SUA) were quantified using an automatic biochemical analyzer (Au5800, Beckman Coulter, USA) and standard enzymatic methods. The estimated glomerular filtration rate (eGFR) was calculated using the MDRD formula as follows: eGFR (mL/min/1.73 m^2^) = 175× (SCr µmol/L/88.402)-1.154 × age-0.203 × (0.742 for females) [28].

### 2.4. Ultrasonographic assessment

A skilled sonographer evaluated the participants with an ACUSON Sequoia ultrasound system (Siemens Medical Systems, Inc., USA) and was blinded to all other data. The participants were examined in the lateral decubitus position. The kidney was initially sectioned longitudinally to determine the maximum thickness from the lower renal pole to the renal fascia, representing the cranial-caudal diameter of perinephric fat. The probe was subsequently positioned at a 90-degree angle to ascertain the transverse and anteroposterior diameters of perinephric fat in the cross-section. The three diameters were ultimately multiplied to calculate the perinephric fat volume (PrFV).

### 2.5. Statistical analysis

The participants were categorized into three groups on the basis of PrFV tertiles: Group 1 (≤36.46 cm³), Group 2 (between 36.59 and 70.76 cm³), and Group 3 (>70.76 cm³). Continuous variables are reported as the means ± SDs for normally distributed data and as medians (interquartile ranges) for skewed data; categorical variables are presented as frequencies (%). One-way ANOVA was used to assess differences in normally distributed variables, the Kruskal-Wallis test was used to assess skewed data, and the chi-square test was used to evaluate categorical variables. Univariate linear regression analysis was subsequently conducted to determine the associations between the PrFV and the eGFR with respect to clinical factors. A multifactorial linear regression model was used to assess the correlation between the PrFV and the eGFR. Initially, the PrFV tertile was entered into the model as an ordinal categorical variable, with the lowest tertile serving as a reference. Model 1 was unadjusted, and Model 2 was adjusted for age, sex, current drinking status, BMI, and WC; SBP, DBP, TG, TC, LDL-C, SUA, and FPG (variable selection was based on those with a *p* value < 0.1 in univariate regression analysis, as well as several traditional indicators). Ultimately, these models were also applied to investigate how a 10 cm^3^ increase in the PrFV affects the eGFR. Statistical analyses were conducted using IBM SPSS software, version 25. Statistical significance was set at *p* ≤ 0.05.

## 3. Results

### 3.1. Clinical characteristics of the participants

Table 1 presents the distribution of perinephric fat levels across the 213 Chinese adults enrolled in the study. Among the three groups, the group with the lowest perinephric fat content comprised younger individuals and a significantly greater percentage of women (*p* < 0.001). Nondrinkers were the most prevalent in this group (*p* < 0.001). Significant intergroup differences were observed for the eGFR, systolic blood pressure (SBP), diastolic blood pressure (DBP), waist circumference (WC), body mass index (BMI), body weight, and triglycerides (TG), total cholesterol (TC), low-density lipoprotein (LDL-C), high-density lipoprotein cholesterol (HDL-C), fasting blood glucose (FPG), and serum uric acid (SUA) levels. Individuals with the highest perinephric fat content presented elevated TG, SBP, DBP, WC, BMI, FPG, and SUA levels and body weight (all *p* < 0.001, except SUA *p* = 0.001), alongside a significantly decreased eGFR and HDL-C level (*p* < 0.001 and *p* = 0.028, respectively).

**Table 1.** Characteristics of the study population stratified across PrFV tertiles.

Parameters	Total (213)	Tertile 1 (71)	Tertile 2 (71)	Tertile 3 (71)	*p*
Age (years)	45.81 ± 10.34	40.93 ± 9.98	46.07 ± 9.47	50.42 ± 9.41	<0.001
Female sex, n (%)	86.0 (40.3)	44.0 (62.0)	23.0 (32.4)	19.0 (26.8)	<0.001
Smoking, n (%)	55.0 (25.8)	14.0 (19.7)	22.0 (31.0)	19.0 (26.8)	0.301
Current drinker, n (%)	59.0 (27.7)	8.0 (11.3)	19.0 (26.8)	32.0 (45.1)	<0.001
WC (cm)	91.87 ± 9.30	85.03 ± 7.88	91.61 ± 6.10	98.99 ± 7.97	<0.001
Weight (kg)	78.0 (68.1-85.0)	67.0 (59.2-77.1)	78.3 (71.3-83.0)	83.0 (78.0-90.0)	<0.001
BMI (kg/m^2^)	25.99 (24.29-28.27)	23.70 (21.8-25.5)	25.76 (24.49-27.51)	28.25 (26.51-30.00)	<0.001
SCr (mmol/L)	79.7 (68.3-88.3)	68.1 (60.4-81.3)	79.0 (69.7-87.1)	86.0 (79.7-95.3)	<0.001
eGFR (mL/min/1.73 m^2^)	83.6 (75.6-92.7)	94.9 (90.5-101.2)	86.7 (80.4-95.6)	87.1 (76.8-94.6)	<0.001
SBP (mmHg)	130 (119-139)	122 (112-131)	132 (123-140)	137 (126-143)	<0.001
DBP (mmHg)	82 (74-90)	74 (68-82)	83 (77-91)	89 (81-94)	<0.001
TG (mmol/L)	1.37 (1.05-2.12)	1.06 (0.87-1.43)	1.58 (1.14-2.16)	1.82 (1.2-2.39)	<0.001
TC (mmol/L)	5.02 (4.36-5.67)	4.54 (3.96-5.45)	5.11 (4.56-5.59)	5.27 (4.46-5.77)	0.023
LDL-C (mmol/L)	3.12 (2.48-3.71)	2.74 (2.23-3.56)	3.23 (2.79-3.78)	3.22 (2.49-3.71)	0.015
HDL-C (mmol/L)	1.34 (1.19-1.54)	1.43 (1.26-1.57)	1.33 (1.18-1.53)	1.27 (1.14-1.47)	0.028
FPG (mmol/L)	5.19 (4.88-5.55)	5.05 (4.72-5.35)	5.23 (4.84-5.53)	5.39 (5.02-6.18)	<0.001
SUA (μmol/L)	370.0 (304.0-424.0)	318.0 (282.0-396.0)	388.0 (318.0-428.0)	397.0 (329.0-442.0)	0.001

Data are expressed as the mean ± SD, median (interquartile range) or number (percentage).

Abbreviations: PrFV, perirenal fat volume; BMI, body mass index; WC, waist circumference; SBP, systolic blood pressure; DBP, diastolic blood pressure; TC, total cholesterol; TG, triglycerides; LDL-C, low-density lipoprotein; HDL-C, high-density lipoprotein; FPG, fast plasma glucose; SCr, serum creatinine; eGFR, estimated glomerular filtration rate; SUA, serum uric acid.

Using the Kruskal-Wallis test and analysis of variance (ANOVA) for continuous variables, the chi-square test for categorical variables.

### 3.2. Scatter plot

**Figure 1.**
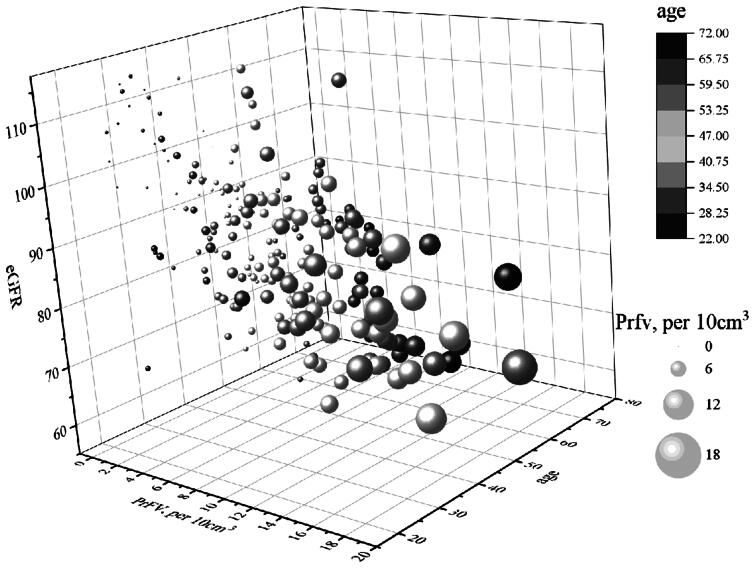
Scatter plot between eGFR with age and PrFv. Abbreviations: PrFV, perirenal fat volume; eGFR, estimated glomerular filtration rate. .

Figure 1 depicts a three-dimensional scatter plot elucidating the relationships among the eGFR, age, and the PrFV. The scatter plot reveals a discernible trend: an increase in the PrFV is associated with a corresponding decrease in the eGFR. This observation implies a potential negative influence of renal adipose tissue accumulation on renal function, potentially diminishing the kidney’s capacity to clear creatinine. Additionally, the scatter plot indicates a decline in kidney function with increasing age. Figure 1 offers an intuitive visualization of the intricate relationships linking creatinine clearance, age, and perinephric fat volume.

### 3.3. Univariate and multivariate analyses

The results of the univariate analysis (Table 2) revealed the predictors of eGFR decline, including age (β: −0.637, *p* < 0.001), SBP (β: −0.183, *p* = 0.003), DBP (β: −0.329, *p* < 0.001), FPG (β: −3.977, *p* = 0.001), WC (β: −0.543, *p* < 0.001), PrFV, per 10 cm^3^ (β: −1.985, *p* < 0.001), BMI (β: −1.489, *p* < 0.001), TC (β: −1.806, *p* = 0.045), LDL-C (β: −2.429, *p* = 0.024) and SUA (β: −0.042, *p* < 0.001). Creatinine clearance tended to decrease as these variables increased.

**Table 2.** Univariate regression analysis for eGFR.

Variables	R^2^	β (95% CI)	*p*
Sex	0.01	1.83 (−1.74−5.41)	0.313
Smoking	0.00	−0.90 (−4.91−3.12)	0.66
Current drinker	0.01	−3.01 (−6.96−0.84)	0.124
Age (years)	0.25	−0.64 (−0.78−0.49)	<0.001
SBP (mmHg)	0.04	−0.18 (−0.30−0.06)	0.003
DBP (mmHg)	0.08	−0.33 (−0.48−0.18)	<0.001
FPG (mmol/L)	0.04	−3.40 (−6.39−1.57)	0.001
WC (cm)	0.15	−0.54 (−0.72−0.37)	<0.001
PrFV (cm^3^), per 10cm^3^	0.29	−1.99 (−2.40−1.57)	<0.001
BMI (kg/m^2^)	0.15	−1.49 (−1.97−1.01)	<0.001
TG (mmol/L)	0.00	−0.67 (−1.80−0.47)	0.248
TC (mmol/L)	0.01	−1.81 (−3.57−0.04)	0.045
LDL-C (mmol/L)	0.02	−2.43 (−4.53−0.33)	0.024
HDL-C (mmol/L)	0.00	2.55 (−3.41−8.50)	0.4
SUA (μmol/L)	0.07	−0.04 (−0.06−0.02)	<0.001

Abbreviations: PrFV, perirenal fat volume; BMI, body mass index; WC, waist circumference; SBP, systolic blood pressure; DBP, diastolic blood pressure; TC, total cholesterol; TG, triglycerides; LDL-C, low-density lipoprotein; HDL-C, high-density lipoprotein; FPG, fast plasma glucose; eGFR, estimated glomerular filtration rate; SUA, serum uric acid; CI, confidence interval.

Table 3 shows that multivariate linear regression analysis revealed a negative linear relationship between the PrFV and eGFR when the PrFV was treated as continuous variable (β: −1.30; 95% CI: −1.87 to −0.74). When the PrFV was analyzed as a categorical variable using tertiles, with tertile 1 as a reference, a negative linear relationship with the eGFR was still observed for tertile 3 (β: −6.91; 95% CI: −11.50 to −2.32). The *p* values for the relationship between the PrFV and eGFR were consistent for both the unadjusted and adjusted models (*p* < 0.001), regardless of whether the PrFV was treated as a continuous or categorical variable.

**Table 3.** The association between PrFV and eGFR.

Variables	R^2^	Model 1 β (95% CI)	R^2^	Model 2 β (95% CI)
Tertiles of PrFV.cm^3^	0.25		0.48	
Tertile 1		Reference		Reference
Tertile 2		−6.65 (−10.38−2.92)**		−1.36 (−5.02−2.30)
Tertile 3		−15.85 (−19.58−12.12)***		−6.91 (−11.50−2.32)**
*p*		<0.001		<0.001
PrFV (cm^3^) per 10cm^3^	0.29	−1.99 (−2.40−1.57)***	0.50	−1.30 (−1.87−0.74)***

Model 1: Unadjusted.

Model 2: Adjusted for age, sex, current drinker, body mass index, waist circumference, systolic blood pressure, diastolic blood pressure, triglycerides, total cholesterol, low-density Lipoprotein, serum uric acid and fast plasma glucose.

Abbreviations: PrFV, perirenal fat volume; CI, confidence interval.

^***^ *p* < 0.001;^ **^p < 0.01.

In the subgroups with and without hypertension, the negative correlation between the PrFV and eGFR remained statistically significant even after adjustment for multiple variables. As shown in Table 4, after multivariate adjustment, the negative correlation between the PrFV and eGFR was stronger in hypertensive group (β: −1.98; 95% CI: −3.13 to −0.84) than in nonhypertensive group (β: −1.15; 95% CI: −1.84 to −0.46).

**Table 4.** The association between PrFV and eGFR according to hypertension.

	Variables	eGFR					
		R^2^	β (95% CI)^a^	*p*	R^2^	β (95% CI)^b^	*p*
Non-Hypertension	PrFV (cm^3^), per 10 cm^3^	0.27	−1.94 (−2.47−1.42)	<0.001	0.50	−1.15 (−1.84−0.46)	0.001
		0.24			0.48		
	Tertile 1		Reference			Reference	
	Tertile 2		−8.63 (−12.98−4.27)	<0.001		−0.96 (−5.30−3.39)	0.663
	Tertile 3		−15.66 (−20.30−11.03)	<0.001		−6.59 (−12.06−1.13)	0.018
Hypertension	PrFV (cm^3^), per 10 cm^3^	0.28	−2.05 (−2.83−1.27)	<0.001	0.45	−1.98 (−3.13−0.84)	0.001
		0.21			0.40		
	Tertile 1		Reference			Reference	
	Tertile 2		0.25 (−9.30−9.81)	0.958		−3.30 (−12.23−5.62)	0.46
	Tertile 3		−12.07 (−21.40−2.74)	0.012		−11.10 (−20.96−1.23)	0.028

^a^ Model 1: Unadjusted.

^b^ Model 2: Adjusted for age, sex, current drinker, body mass index, waist circumference, triglycerides, total cholesterol, low-density Lipoprotein, serum uric acid and fast plasma glucose.

Abbreviations: PrFV, perirenal fat volume; CI, confidence interval.

As shown in Table 5, after multivariate adjustment, a significant correlation between the PrFV and the eGFR was observed in both males and females. The negative correlation between the PrFV and the eGFR was weaker in males (β: −1.33; 95% CI: −2.00 to −0.66) than in females (β: −1.63; 95% CI −2.86 to −0.39). Among individuals with hypertension, those without hypertension and males, a significant association was observed between the third PrFV tertile and the eGFR when the first tertile was used as a reference. This association remained significant after multivariate adjustment. However, in females, this association did not reach statistical significance.

**Table 5.** The association between PrFV and eGFR according to sex.

Sex	Variables	eGFR					
		R^2^	β (95% CI)^a^	*p*	R^2^	β (95% CI)^b^	*p*
Male	PrFV (cm^3^), per 10 cm^3^	0.28	−1.84 (−2.36−1.33)	<0.001	0.46	−1.33 (−2.00−0.66)	<0.001
		0.23			0.42		
	Tertile 1		Reference			Reference	
	Tertile 2		−7.81 (−12.98−2.65)	0.003		−1.66 (−6.74−3.42)	0.518
	Tertile 3		−15.71 (−20.80−10.62)	<0.001		−7.29 (−13.26−1.31)	0.017
Female	PrFV (cm^3^), per 10 cm^3^	0.47	−3.07 (−3.76−2.37)	<0.001	0.57	−1.63 (−2.86−0.39)	0.010
		0.37			0.55		
	Tertile 1		Reference			Reference	
	Tertile 2		−8.09 (−13.71−2.47)	0.005		−0.19 (−5.78−5.40)	0.946
	Tertile 3		−21.82 (−27.82−15.83)	<0.001		−7.16 (−15.36−1.05)	0.086

^a^ Model 1: Unadjusted.

^b^ Model 2: Adjusted for age, current drinker, body mass index, waist circumference, systolic blood pressure, diastolic blood pressure, triglycerides, total cholesterol, low-density Lipoprotein, serum uric acid and fast plasma glucose.

Abbrviations: PrFV, perirenal fat volume; CI, confidence interval.

## 4. Discussion

The criteria for chronic kidney disease (either of the following present for a minimum of 3 months) include: the presence of markers of kidney damage (1 or more), i.e., 1. albuminuria (an albumin-to-creatinine ratio (ACR)≥30 mg/g), 2. urine sediment abnormalities, 3. persistent hematuria, electrolyte and other abnormalities due to tubular disorders, 4. structural abnormalities detected by histological analysis or imaging, and 5. a history of kidney transplantation, and a decreased GFR, defined as a GFR <60 mL/min per 1.73 m^2^ [2]. This study focused on individuals undergoing health screening, aiming to thoroughly investigate relevant medical issues. All participants were rigorously selected on the basis of established inclusion and exclusion criteria to ensure the scientific validity and representativeness of the study sample. In accordance with the guidelines outlined in the KDIGO 2024 Clinical Practice Guideline for the Evaluation and Management of Chronic Kidney Disease, individuals at risk of chronic renal failure were explicitly excluded from this study. This screening strategy not only effectively reduced the potential incidence of chronic renal failure among the study subjects but also strongly guaranteed the accuracy and reliability of the study results.

During the study design phase, to ensure the rational use of social resources, we did not measure the urinary albumin-to-creatinine ratio (ACR) of the participants or perform urinary sediment analysis. This decision was based on the recommendation of the KDIGO 2024 Clinical Practice Guideline for the Evaluation and Management of Chronic Kidney Disease, which states that testing for chronic renal failure should be prioritized for high-risk populations. Given that the study subjects were individuals with a relatively low incidence of chronic renal failure who were undergoing a health screening, the aforementioned tests were not performed.

In clinical research, the use of computed tomography (CT) and magnetic resonance imaging (MRI) for the measurement of perirenal fat is limited by several factors, including long examination times, radiation exposure risks, high equipment costs, sensitivity to metallic implants, low reproducibility, and restricted applicability to certain populations. In contrast, ultrasound measurements of perirenal fat yield significant advantages, including ease of operation, efficiency, low cost, high reproducibility, and the absence of ionizing radiation. These benefits make ultrasound particularly suitable for specific populations, such as pregnant women, children, and patients with impaired renal function. Ultrasound can provide real-time visualization of the morphology and thickness of perirenal fat and its spatial relationships with surrounding tissues, facilitating dynamic observation and real-time measurement. Its real-time imaging capability further enhances the flexibility and reproducibility of ultrasound in the assessment of perirenal fat. In recent years, ultrasound has been widely adopted in numerous clinical studies to measure perirenal fat, with the aim of evaluating its associations with renal function, atherosclerosis, lipid levels, serum uric acid levels, and other clinical indicators [26,28–30].

In this study, ultrasonography was used to measure the perirenal fat volume. However, this method is limited by technical challenges for individuals weighing more than 80 to 100 kilograms. In obese individuals, the increased thickness of subcutaneous fat limits the penetration of ultrasound waves, reduces image resolution, and obscures the details of perirenal fat. Despite these limitations, ultrasonography was chosen as the measurement method. This decision was based on a study in which more than 60% of participants weighed more than 80 kilograms. The inter-operator repeatability assessed by the difference between ultrasonography and CT was only 3.2% [31]. This finding indicates that ultrasonography is a reliable and reproducible technique for measuring perirenal fat volume, even in obese subjects, and it yields accurate results.

This study is the first to investigate the relationship between the ultrasound-measured PrFV and estimated eGFR in a cohort of Chinese adults undergoing health screenings. Our findings demonstrate that the PrFV is significantly inversely associated with the eGFR, suggesting an independent relationship.

Multiple investigations have investigated the correlation between perinephric fat and the eGFR among individuals with hypertension and/or type 2 diabetes. Lamacchia et al. [32] reported an association between a greater perinephric fat thickness measured *via* ultrasound and a decreased eGFR in a cohort of 151 type 2 diabetic patients, with adjustments made for established risk factors such as BMI and WC. Fang et al. corroborated these findings, noting a significant negative correlation between perinephric fat thickness and the eGFR in male type 2 diabetic patients, which implies a potential role for perinephric fat in the etiology of renal dysfunction in this population [28]. Geraci et al. reported a negative correlation between the thickness of perirenal and perinephric fat and the eGFR in hypertensive patients [24]. Our study confirmed that, in individuals without hypertension, the PrFV is significantly and negatively correlated with the eGFR. After univariate analysis, we found that the eGFR was significantly correlated with BMI and the WC, which are common clinical indicators of obesity, consistent with previous studies [10,25,33]. Upon adjustment for PrFV, no correlation was identified between the WC, BMI and the eGFR, a result that is in accordance with Lamacchia et al.’s study. In contrast to prior studies, our investigation was predominantly conducted on individuals undergoing health screenings. An extensive review of the literature revealed that the majority of studies focusing on perinephric fat have focused on diabetic and hypertensive populations. Our study focused on the relationship between perirenal fat volume and the eGFR during a population health screening, but the results were consistent with those of previous studies, confirming a negative correlation between the perinephric fat volume and the eGFR.

Following adjustments for potential confounders such as BMI, the WC, alcohol consumption, and sex, the negative correlation between the PrFV and eGFR remained significant. However, a study on a population without hypertension and diabetes reported no relationship between the perinephric fat thickness and the eGFR [31]. This study revealed that perirenal fat is significantly associated with the eGFR in both males and females, with a more pronounced correlation observed in females. This finding is consistent with the study by Xu et al. [25]. However, another study indicated that the eGFR is significantly negatively correlated with perirenal fat in the overall population and in males, but no significant association was observed in females [28]. This discrepancy may arise from differences in the measurement methods used for perirenal fat and the characteristics of the study populations. As previously noted, perinephric fat distribution is heterogeneous, with the greatest concentration in the lower perinephric region. Consequently, we employed the volume of perinephric fat in the lower region determined by ultrasound as a quantitative indicator.

The intimate association between perinephric adipose tissue (PRAT) and renal function, which is attributed to their spatial proximity and shared neural and vascular networks, is believed to substantially influence kidney function, particularly in the context of PRAT expansion, dysfunction, and inflammation [34,35]. While the precise mechanisms underlying the role of PRAT in the induction and exacerbation of chronic kidney injury remain unclear, several plausible mechanisms have been proposed to account for the link between PRAT and chronic kidney disease. Initially, the accumulation of PRAT could result in direct compression of the renal parenchyma and vasculature. Consequently, this compression may increase sodium reabsorption, increase blood pressure, and alter renal function [36]. An abundance of PRAT encircling the kidney can increase interstitial hydrostatic pressure within the renal parenchyma, thereby diminishing renal blood flow and contributing to increased sodium reabsorption and activation of the renin-angiotensin system (RAS). These processes can accelerate the progression of kidney disease, ultimately leading to a decrease in the glomerular filtration rate (GFR) [35–37]. Second, an overabundance of PRAT is positively associated with increased free fatty acid (FFA) secretion, which can precipitate chronic inflammation, a hallmark of obesity [23]. FFA metabolites exert direct nephrotoxic effects and are significantly associated with albuminuria [38]. Third, excess PRAT may impact renal function *via* the local or systemic release of proinflammatory cytokines, including tumor necrosis factor-a (TNF-a), plasminogen activator inhibitor-1 (PAI-1), and leptin, potentially influencing renal function in a paracrine fashion [27,39–41]. Recent investigations in nonobese prediabetic rats have demonstrated that localized PRAT inflammation, which is marked by the upregulation of interleukin-1β (IL-1β), can trigger structural and functional renal deterioration linked to alterations in vascular endothelial function [42].

The authors of this study acknowledge several limitations. First, the study population was a single-center cohort with homogeneous racial demographics, which may limit the generalizability of the findings to diverse racial groups. Second, our study was cross-sectional, and the causal relationship between the PrFV and the eGFR could not be determined. Third, the study did not account for potential confounders related to the GFR, including dietary and lifestyle factors, because of the absence of relevant data for this population. Future validation of this correlation could be achieved through prospective cohort studies or interventional studies.

## 5. Conclusion

In conclusion, our study demonstrates a significant inverse relationship between the perinephric fat volume (PrFV) and the estimated glomerular filtration rate (eGFR), warranting further investigation into the mechanistic role of perinephric fat in CKD.

## Data Availability

The dataset generated and analyzed in this study can be obtained from the authors.
